# The Synergistic Reduction of the Contact Time in the Droplet Impact on a Moving Ridge Surface

**DOI:** 10.34133/research.0543

**Published:** 2024-12-09

**Authors:** Jiayi Zhao, Wenlong Yu, Wenhao Wang, Shuo Chen, Diangui Huang

**Affiliations:** ^1^ School of Energy and Power Engineering, University of Shanghai for Science and Technology, Shanghai 200093, China.; ^2^ School of Aerospace Engineering and Applied Mechanics, Tongji University, Shanghai 200092, China.

## Abstract

The contact time of the droplet impacting on solid surfaces can be markedly reduced by 40% to 50% by breaking the symmetric behaviors with the help of the surface structures and motion, which is crucial to diverse applications involving anti-icing, anti-erosion, self-cleaning, etc. Herein, it is interesting to note that the contact time can be further decreased up to 60% on a moving ridge surface because of corresponding synergy, inspired by flying insects or wind-dispersal seeds. In the present work, the synergistic mechanisms of the reduction in contact time have been revealed by analyzing the 3 basic features, called Leaf-type, Ear-type, and Butterfly-type, according to their morphological and dynamical behaviors. Therefore, a universal theoretical model has arrived by introducing normal and tangential Weber numbers, beyond previous descriptions. Importantly, our study discovers a generalized scaling law of −0.52 between the contact time and new composite Weber number (*We_com_*), which is feasible to stationary and moving surfaces, suggesting that the limit reduction rate on a moving ridge surface tends to 78%. The present work provides an insight to optimize the corresponding application efficiency by coupling the surface structure and motion.

## Introduction

Droplet impacts on a solid surface is ubiquitous in nature and of practical importance in various engineering applications, such as rain drop impact [1,2], inkjet printing and coating [3], and pesticide splashing [4], which has continued to attract considerable attention in recent years. These applications mainly rely on the spreading ability of the droplet. Besides, a pave way to obtain the shorter contact time is also an important concern, which has contributed to drag reduction [5,6], staying dry [7], self-cleaning [8,9], and anti-icing [10,11]. Weakening the solid–liquid contact and promoting the rapid detachment of the droplet from surfaces are the primary method to achieve enhanced effect in these application scenarios. Therefore, the ways to shorten the contact time are essential and have attracted increasing attention.

The contact time *t_c_* is defined as the time interval between first touching and completely bouncing off the substrate. *t_c_* scales with the inertial capillary time scale *τ*_0_ = (*ρR*_0_^3^/*σ*)^0.5^, independent of the impact velocity. Here, *ρ*, *R*_0_, and *σ* represent the mass density, radius, and surface tension of the liquid droplet, respectively. The dimensionless contact time *t_c_*/*τ*_0_ is measured as 2.6 ± 0.1 by balancing inertia with capillarity during droplet impacts on a static superhydrophobic surface [12].

**Fig. 1.**
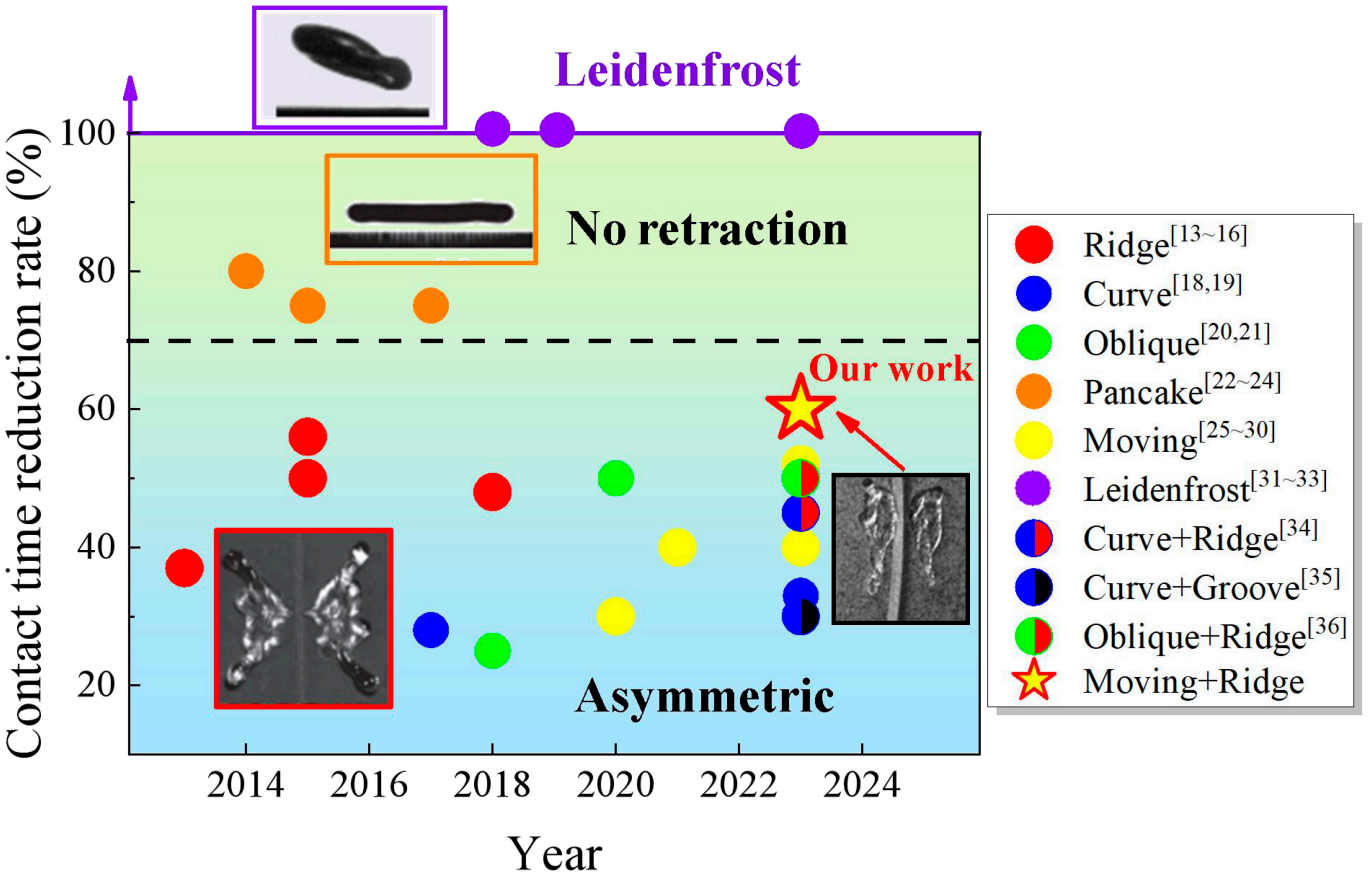
A brief review of the reduction rate of contact time [13–33]. According to the principle of time reduction, these methods can be divided into 3 categories: asymmetric, no retraction, and Leidenfrost effect.

Inspired by surface structures and behavior of natural insects and plants [13–17], breaking the isotropy of the droplet during the impact process is believed to result in the reduction in contact time, involving ridge surfaces [13–16], curved surfaces [18,19], oblique surfaces [20,21], post arrays [22–24], surface movement [25–30], and Leidenfrost [31–33]. Recently, the ways of coupling active and passive controls are verified to further reduce the contact time. In particular, the contact time can be decreased from 30% to 50% max [34–36], as summarized in Fig. 1.

**Fig. 2.**
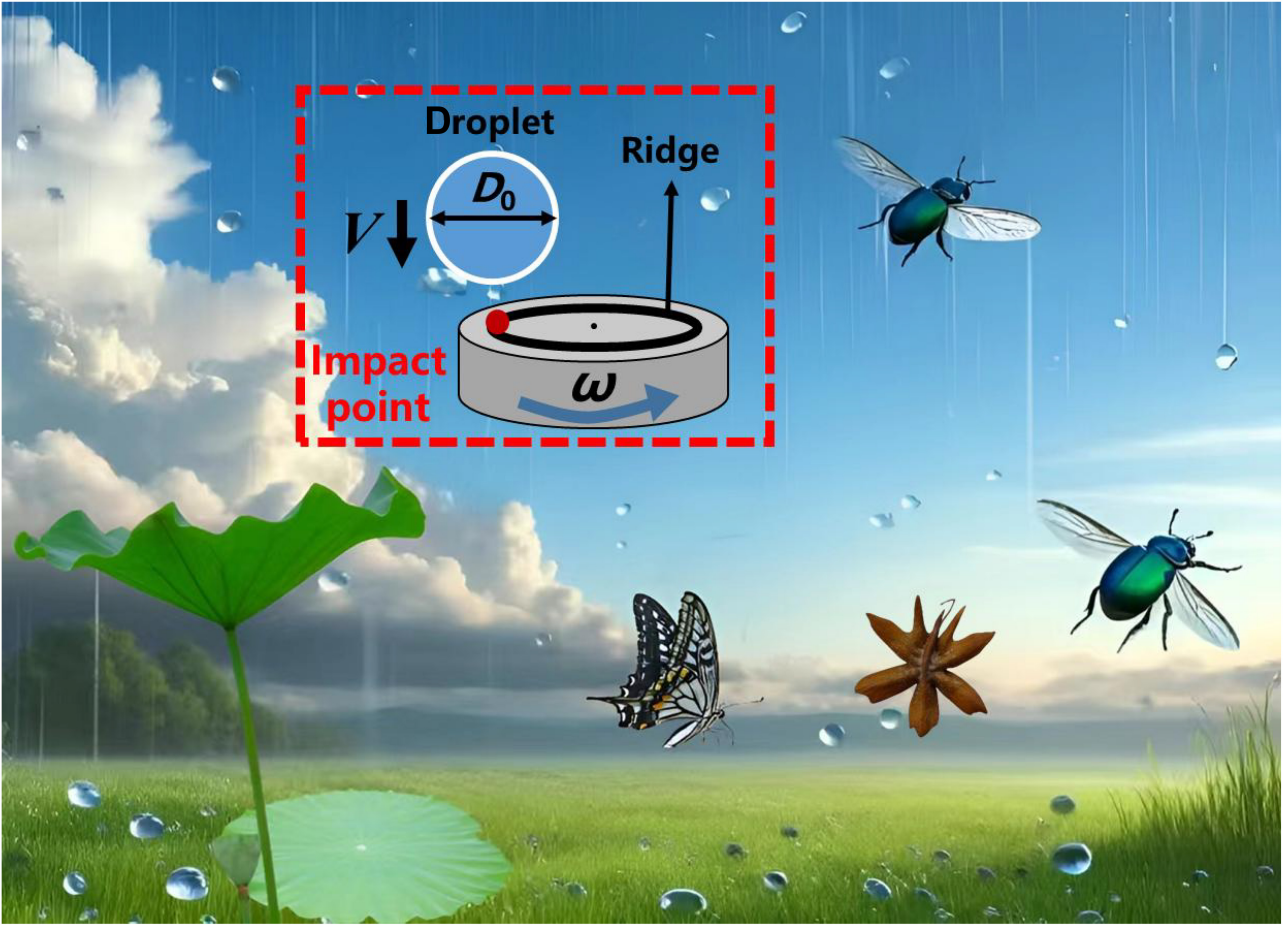
Nature-inspired contact time reduction strategy.

However, the deeper understandings of the synergistic mechanisms in the contact time reduction are required to further optimize the application efficiency. In the present work, the reduction of the contact time is validated successfully to be up to 60% by introducing a moving ridge surface enlightening by the flying insects or wind-dispersal seeds [13–17], which is optimum in asymmetric rebounding to our knowledge. Moreover, we adopt the experiments, numerical simulations, and theoretical models to reveal corresponding synergy morphologically and dynamically, as seen in Fig. 2. The synergistic mechanisms are illustrated by a universal theoretical model in the range of normal (*We_n_* = 14.7 to 71.2) and tangential Weber number (*We_τ_* = 0 to 403.5); meanwhile, a generalized scaling law is reached by using a composite Weber number.

## Results and Discussion

### Experimental system and findings

**Fig. 3.**
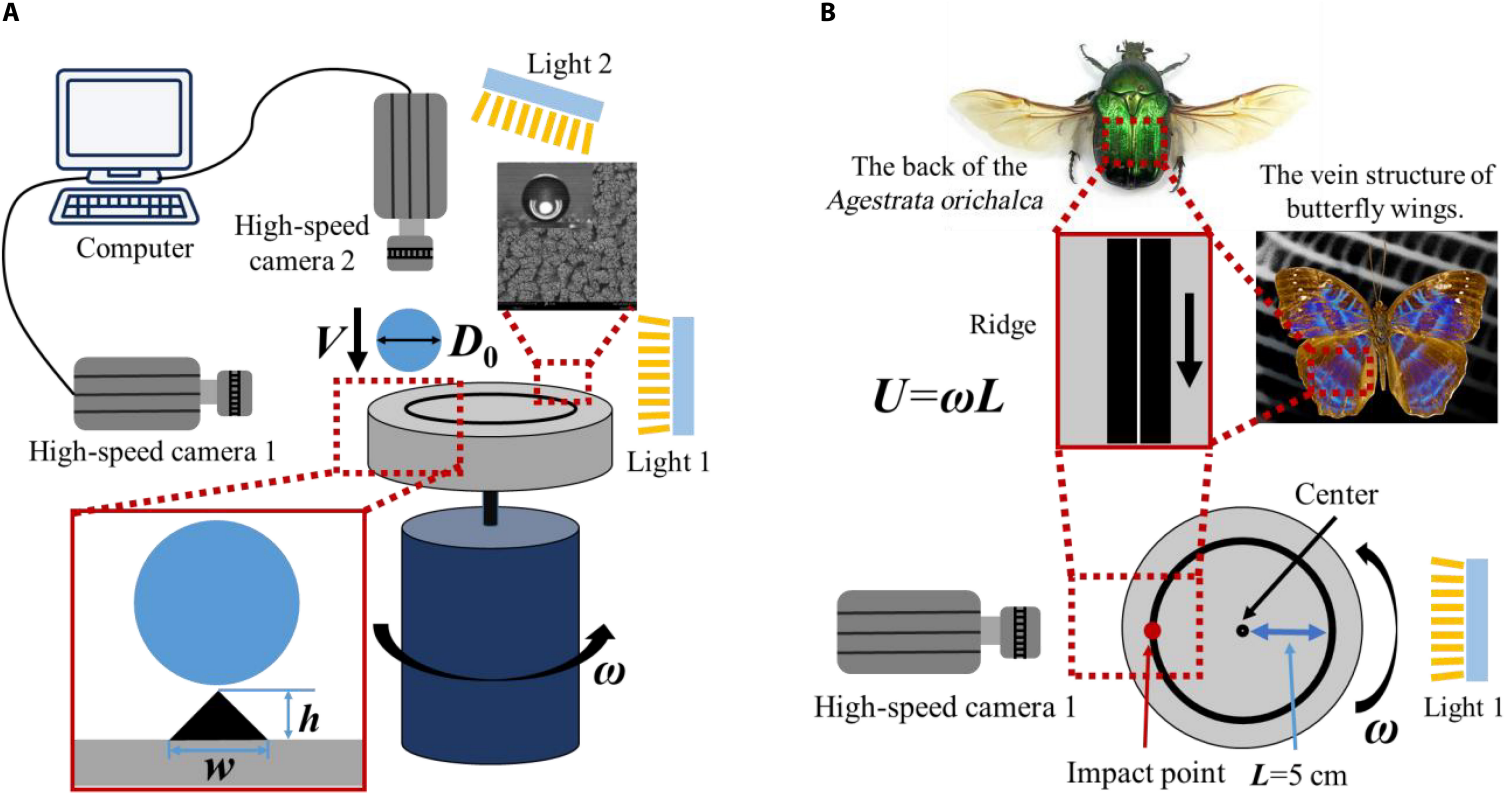
Experimental schematic diagram. (A) Display of experimental equipment and methods in the front view, where *h* = 0.5 mm and *w* = 1 mm. (B) Display of surface and impact points in the top view, with a biomimetic structural diagram.

In this work, the dynamical behavior of the droplet impacting a moving ridge surface is studied, where the normal momentum of the droplet and the tangential momentum of the surface can be well controlled, as shown in Fig. 3A. In particular, the polished Al-based substrate is treated by a commercial agent (NeverWet, Rust-Oleum Corporation, USA) to obtain superhydrophobicity. The contact angle of a deionized water droplet with *D*_0_ = 2.1 ± 0.05 mm is approximately 163° ± 3°, and the contact angle hysteresis reaches 5° ± 2°. The schematic of the present experiment and corresponding scanning electron microscopy image of the surface are indicated in Fig. 2A. Subsequently, the substrate has been entrained by a stepper motor, widely utilized in droplet impingement on moving surfaces [25–28]. The horizontal surface velocity *U* is managed by the rotor revs and measured distance between impact point and disk center, corresponding to *We_τ_* = *ρU*^2^*D*_0_*/σ* = 0 to 403.5. The impact velocity of the droplet is determined by the height of droplet release, and the corresponding Weber number is *We_n_* = *ρV*^2^*D*_0_*/σ* = 14.7 to 71.2 (the impact velocity obtained based on free fall is close to that obtained based on image measurement, with an error of less than 3% between the 2 ways; see Fig. S1). Besides, the curvature of the ridge is much smaller than the droplet diameter, so it can be considered that the ridge moves in a straight line [24] (Fig. 3B).

**Fig. 4.**
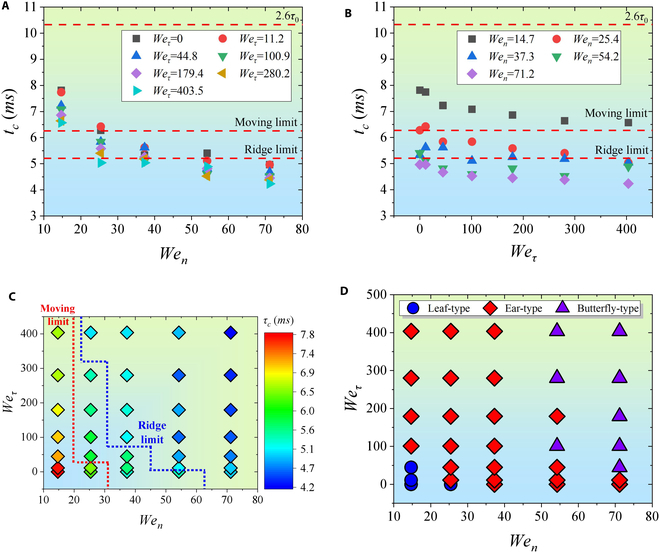
The contact time diagram and dynamic behavior regime map under the experimental results. (A) Relationship between *τ_c_* and *We_n_* under different *We_τ_*. Under the same *We_n_*, the change in *τ_c_* is small. (B) Relationship between *τ_c_* and *We_τ_* under different *We_n_*. Under the same *We_τ_*, the change in *τ_c_* is larger. (C) Two-dimensional distribution of *τ_c_* with respect to *We_n_* and *We_τ_*. An increase in *We_n_* and (or) *We_τ_* will lead to a reduction in *τ_c_*. (D) Regime map of the droplet shape at maximum normal spreading moment. Different shapes can affect the behavior of the droplet during the retraction process.

Figure 4A and B illustrates the contact time on droplet impact on the moving ridge surface, and the contact time is less than 2.6*τ*_0_ [12]. It can be seen that the contact time of the droplet impacting the static ridge (*We_τ_* = 0) is reduced to 1.3*τ*_0_, which is consistent with the conclusion of Gauthier et al. [14]. Most of the contact time is shorter than 1.56*τ*_0_, which is obtained on the moving surface according to Zhang et al. [27]. Figure 4C reflects the evolution of the contact time with respect to *We_τ_* and *We_n_*. The contact time generally decreases with the increase of *We_τ_* and *We_n_*, where *We_n_* has a stronger effect. Finally, the post-impact behaviors can be divided into 3 types by the shape of the droplet during the impact: Leaf-type, Ear-type, and Butterfly-type (Fig. 4D). Obviously, the division of these types depends on the change of Weber numbers and effectively affects the contact time.

To gain insights into the physical mechanisms underlying the observed contact time reduction, we further analyze the impact dynamics of the 3 types.

### Experimental characterization and theoretical analysis

#### Leaf-type: Tangential retraction behavior

**Fig. 5.**
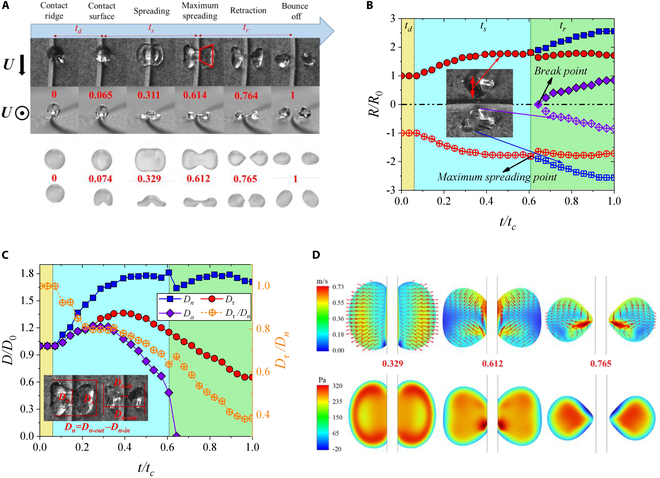
Evolutionary behavior of Leaf-type with *We_τ_* = 11.2 and *We_n_* = 14.7. (A) Snapshots and numerical simulation results of the droplet. The top and main views of the experimental snapshots, and the simulated top and main view results are presented from top to bottom, respectively. The process of droplet impact can be divided into 3 stages: dropping (*t_d_*), spreading (*t_s_*), and retraction (*t_r_*). These red numbers represent dimensionless time, and *U* is the direction of movement for the ridge. (B) Evolutions of normal spreading radius. The red dot represents the normal radius of the droplet. The blue dot represents the vertical distance from the outer edge of the droplet to the center of the ridge, and the purple dot shows the vertical distance from the inner edge of the droplet to the center of the ridge. *t_c_* is the contact time. (C) Evolutions of spreading diameters. *D_n_*, *D_τ_*, *D_a_*, and *D_τ_*/*D_n_* have been labeled. (D) Velocity and pressure distribution at a droplet cross-section 0.3 mm away from the surface. The corresponding times from left to right are *t*/*t_c_* = 0.329, 0.612, and 0.765 (during the spreading process, the maximum spreading moment, during the retraction process), respectively. The experimental and simulation videos can be seen in Movie S1.

Figure 5A shows the experimental and numerical dynamical behavior for Leaf-type in front and top views under *We_τ_* = 11.2 and *We_n_* = 14.7. For Leaf-type, droplet spreads rapidly in the normal direction (perpendicular to the ridge) due to the ridge cutting and low *We_τ_* after contacting the surface. During the normal spreading, droplet spreads to the tangential maximum spreading diameters (parallel to the ridge) and then starts to retract tangentially. After reaching the maximum normal spreading, the droplet continues to retract tangentially until it bounces off the surface. We further obtain the temporal evolution of droplet normal spreading length *D_n_*, tangential spreading length *D_τ_*, and the spreading length on the ridge *D_a_*, normalized by its initial diameter *D*_0_, as shown in Fig. 5B and C. Figure 5B shows the temporal evolution of the spreading radius (*R_n_*, *R_in_*, and *R_out_*; the corresponding diameters are shown in Fig. 5C). It can be found that the impact behavior can be divided into 3 stages: dropping, spreading, and retraction. In the dropping stage, the droplet diameter remains unchanged. In the spreading stage, the droplet spreads to its maximum normal radius and then breaks into 2 subdroplets. After breaking, the inner (purple dots) and outer (blue dots) edges of the subdroplets move away from the ridge at the same time but retain roughly the same normal radius (red dots). For the evolution of holistic spreading length, it can be seen from Fig. 5C. Normal spreading is obviously stronger than tangential spreading, and spreading also exists on the ridge. The subdroplets then begin to retract tangentially on the ridge. Because tangential spreading and retraction are out of sync with normal spreading and retraction, the ratio of tangential and normal length (*D_τ_*/*D_n_*) decreases from 1 to 0.4, leading to a long shape for droplet perpendicular to the ridge monolithically.

To further explore the velocity and pressure distribution of the droplet, we conducted the numerical simulation of the droplet using the commercial fluid simulation software COMSOL Multiphysics (version 6.1). From the velocity fields and pressure fields (Fig. 5D), we can find that during the spreading stage, the droplet maintains a uniform normal spreading velocity as a whole. At the maximum spreading moment, the normal spreading velocity at the outer edge of the droplet tends to 0, while the interior keeps high tangential retraction velocity and low normal spreading velocity. During the retraction stage, the droplet still maintains a spreading trend in the normal direction, but the tangential retraction velocity is more pronounced, leading to the tangential retraction behavior. From the perspective of pressure field, the variations in pressure are minimal due to the low values of *We_τ_* and *We_n_*. This results in the maintenance of a low-pressure region surrounding a high-pressure area at the center during the impact progress.

#### Ear-type: Normal retraction behavior affected by breaking

**Fig. 6.**
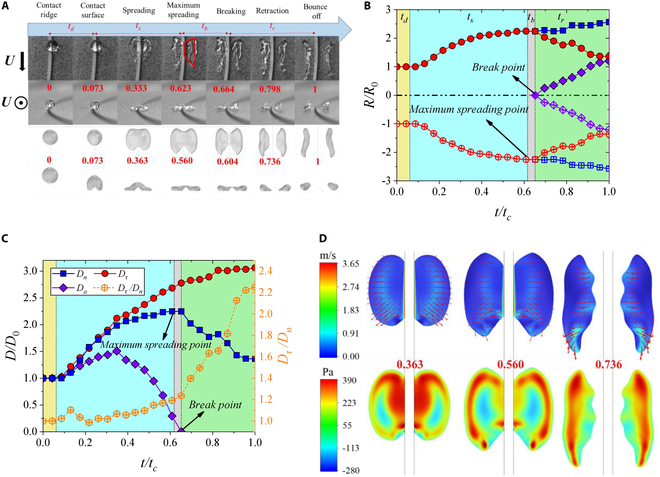
Evolutionary behavior of Ear-type with *We_τ_* = 403.5 and *We_n_* = 37.3. (A) Snapshots and numerical simulation results of the droplet. The process of droplet impact can be divided into 4 stages: dropping (*t_d_*), spreading (*t_s_*), breaking (*t_b_*), and retraction (*t_r_*). (B) Evolutions of normal spreading radius. (C) Evolutions of spreading diameters. (D) Velocity and pressure distribution. The experimental and simulation videos can be seen in Movie S2.

Similar to the Leaf-type impact behavior, under the Ear-type impact behavior, the droplets also undergo 3 distinct stages: dropping, spreading, and retraction. However, there are notable differences between the 2 behaviors. Figure 6A shows the experimental and simulated dynamical behavior for Ear-type when *We_τ_* = 403.5 and *We_n_* = 37.3. The droplet drops onto the surface after contacting the ridge and then starts spreading until maximum normal spreading. Subsequently, the droplet begins to retract normally until it bounces off, during which it breaks into 2 subdroplets. During the normal retraction stage, the droplet continuously spreads in the tangential direction due to the high *We_τ_*. From the evolution of normal radius (Fig. 6B), the break point is later than the maximum spreading point, which causes asynchronous retraction of the inner and outer edges of the droplet. In addition, unlike Leaf-type, the normal diameter (red dots) gradually decreases after the maximum spreading point. So, the combined effect of tangential spreading and normal retraction causes the broken subdroplets to stretch into long strips parallel to the ridge. In Fig. 6C, we can also find that the droplet is continuously spreading in the tangential direction and is stronger than the normal spreading. *D_τ_*/*D_n_* increases from 1 to 2.4, consistent with the long strips of droplets.

Compared to the velocity of the ridge, the spreading and retraction speeds of the droplet are smaller (Fig. 6D). During the spreading stage, the droplet maintains a uniform normal spreading while maintaining a fast tangential spreading speed along the leading edge of the ridge's movement direction. At the maximum normal spreading point, the normal spreading speed toward the outer edge tends to 0, while the internal tangential and normal spreading are weak. During the retraction stage, the normal spreading stops at the outer edge, while the interior continues to retract toward the outer edge. At the same time, the tangential spreading speed of the leading edge further increases. Besides, a high *We_τ_* reduces the pressure at the leading edge of the droplet, while the high-pressure zone diffuses from the center to the outer edge, resulting in an increase in the velocity of the corresponding low-pressure zone.

#### Butterfly-type: Normal retraction behavior not affected by breaking

**Fig. 7.**
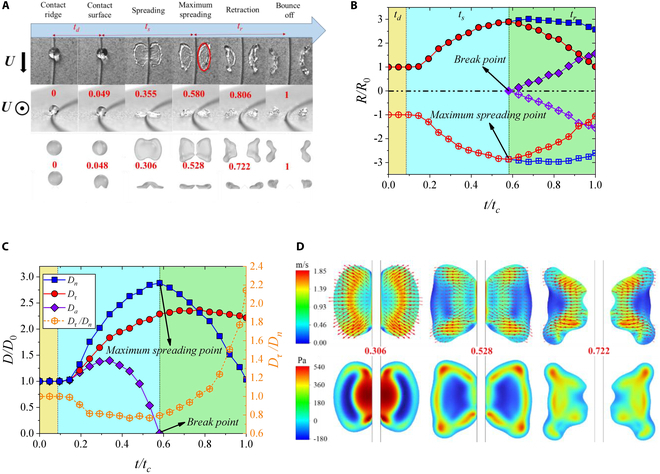
Evolutionary behavior of Butterfly-type with *We_τ_* = 100.9 and *We_n_* = 71.2. (A) Snapshots and numerical simulation results of droplets. The process of droplet impact can be divided into 3 stages: dropping (*t_d_*), spreading (*t_s_*), and retraction (*t_r_*). (B) Evolutions of normal spreading radius. (C) Evolutions of spreading diameters. (D) Velocity and pressure distribution. The experimental and simulation videos can be seen in Movie S3.

For Butterfly-type, we analyze the results as *We_τ_* = 100.9 and *We_n_* = 71.2. It can be found that the impacting can also be divided into 3 stages (Fig. 7A), same with Leaf-type. Compared with Ear-type, the break point is earlier than the maximum spreading point, which makes the normal retraction behavior unaffected by breaking of the droplet. Due to the impact at the center of the ridge, the evolution of the spreading radius is also axisymmetric, seen in Fig. 7B. Meanwhile, the increase in *We_n_* strengthens the cutting of the droplet by the ridge and also increases the dimensionless radius of the inner edge of the droplet (*R_in_*/*R*_0_) at the moment of bouncing off from 0.9 (purple dots in Fig. 5B) for Leaf-type and 1.2 (purple dots in Fig. 6B) for Ear-type to 1.7 for Butterfly-type (purple dots). Figure 7C indicates that the droplet first spreads and then retracts in tangential and normal directions, but the tangential speed of spreading and retraction is smaller than that of normal spreading and retraction. Therefore, the ratio of tangential and normal diameter (*D_τ_*/*D_n_*) decreases from 1 to 0.8 and then increases to 2.2.

From the simulation results of Fig. 7D, it can be seen that the droplet maintains a uniform normal spreading speed, and the overall velocity direction is emission-like during the spreading stage. The normal spreading speed toward the outer edge tends to 0, while the internal tangential and normal spreading speed are low at the point of maximum normal spreading. However, the spreading speed at the 4 corners is relatively high, causing the 4 lobes. Then, the outer edge stops spreading in the normal direction, while the interior retracts in the normal direction toward the outer edge, and the raised corners further spread during the retraction stage. On the other hand, a higher *We_n_* reduces the pressure at the center, while the high-pressure zone diffuses to the surrounding area, resulting in an increase in velocity in the 4 corners.

#### Synergistic mechanism: Contact time reduction

To investigate the change of contact time on the moving ridge surface, we first divide the droplet impact behavior into several stages and study them separately, as mentioned earlier. There are 3 essential phases as dropping, spreading, and retraction regardless of the rebounding types. In addition, there is a breaking stage after the maximum spreading point of Ear-type. The time between the droplet contacts the ridge and the surface is defined as the dropping stage, and it can be considered that the falling speed of the droplet is constant, i.e., *V*. So, the drop time *t_d_* can be obtained, i.e., *t_d_* = *h*/*V*, where *h* is the height of the ridge.

In the spreading stage, the droplet begins to spread until it reaches the maximum normal spreading. The proportion of the spreading time *t_s_* to the contact time *t_c_* remains stable at 0.584, i.e., *t_s_*/*t_c_* = 0.584 (Fig. S2), which can be found through a large amount of experimental data.

In the retraction stage, due to the different ways of retraction, the retraction times *t_r_* for 3 types of impact behaviors will be discussed separately below. For tangential retraction of Leaf-type, the retraction time *t_r_* depends on the tangential spreading length *D_τ_* at the maximum normal spreading point and the tangential retraction velocity *V_ret_*. At the maximum normal spreading point, *D_τ_* is close to the maximum tangential spreading length of the droplet impacting on the moving surface, as the cutting operation of the ridge. So, *D_τ_* can be approximated through this model proposed by Aboud and Kietzig [20], and the theoretical tangential contact length also fit well with the experimental values (see Fig. S3):

$$D_{\tau }/D_{0}=1.218{\text{We}}_{n}^{0.29}+0.012\sqrt{{\text{We}}_{n}{\text{We}}_{\tau }}$$ (1)

For the maximum normal spreading length *D*_*n-*max_, Li et al. [37] found that the ratio of *D_τ_* to *D_n-max_* in the same time is found to be positively correlated with the ratio of the 2 Weber numbers of the droplet impacting on the moving surface. Based on this conclusion and combined with our experimental data, it is found that the droplet still follows this linear relationship at maximum normal spreading point (see Fig. S4):

$$D_{\tau }/D_{n-\max}=0.11\sqrt{{\text{We}}_{\tau }/{\text{We}}_{n}}+0.73$$ (2)

For water droplet with low viscosity, the viscous effect can be neglected because the impact number *WeRe*^−4/5^ is much smaller than 1 [38]. Thus, the retraction velocity follows the classical Taylor–Culick velocity [39], *V_ret_* ≈ [(1 − cos *θ*) *σ*/*ρh*]^0.5^, where *h* is the average film thickness at the maximum normal spreading. For Leaf-type, the liquid film at the maximum normal spreading is similar to 2 trapeziums (inset in Fig. 5A), and the area of these trapeziums is *S* ≈ [(*D_τ_* + *D_a_*) × (*D_n-max_*/2)/2] × 2 = [(*D_τ_* + *D_a_*) × *D_n-max_*]/2, where *D_a_* is the film length on the ridge at this time. Mass conservation gives *h* = *πD*_0_^3^/6*S*, and thus, *t_r_* can be expressed as:

tr=Dτ2Vret≈Dτ×ρh8σ=ρDτ28σ×πD036S=ρDτ28σ×πD033Dτ+DaDn−max=ρπD0324σ×Dτ2Dτ+DaDn−max (3)

With terms of Ear-type, the retraction of the droplet is affected by the time of breaking, because the breaking occurs during the retraction stage. During the period from the maximum normal spreading point to the breaking point, the outer edge of the droplet begins to retract, but the inner edge does not retract. We define this time as the breaking time *t_b_* of the droplet. Similarly, the retraction velocity *V_ret_* is obtained by the maximum normal spreading point, and the shape of the droplet is also similar to 2 trapeziums (inset in Fig. 6A). During the period of *t_b_*, the retraction length of the outer edge of the droplet can be obtained as *t_b_* × *V_ret_*. After breaking, the inner and outer edges of the droplet retract at the same time until they bounce off the surface, and the retraction length is *D_n-max_* − 2*t_b_* × *V_ret_*. So, *t_r_* can be expressed as:

tr=Dn−max−2tbVret4Vret=Dn−max4Vret−tb2≈ρDn−max232σ×πD036S−tb2=ρDn−max232σ×πD033Dτ+DaDn−max−tb2=ρπD0396σ×Dn−maxDτ+Da−tb2 (4)

Because the breaking point of Butterfly-type is before the maximum normal spreading point, the inner and outer edges of the subdroplets retract at the same time during the retraction stage. At the maximum normal spreading point, the shapes of the subdroplets are 2 ellipses (inset in Fig. 7A), so the area of the 2 ellipses is *S* ≈ 2*π*(*D_τ_*/2) × (*D_n-max_*/4). Similarly, *t_r_* can be obtained as:

tr=Dn−max4Vret≈ρDn−max232σ×πD036S=ρDn−max232σ×2D033DτDn−max=ρD0348σ×Dn−maxDτ (5)

**Fig. 8.**
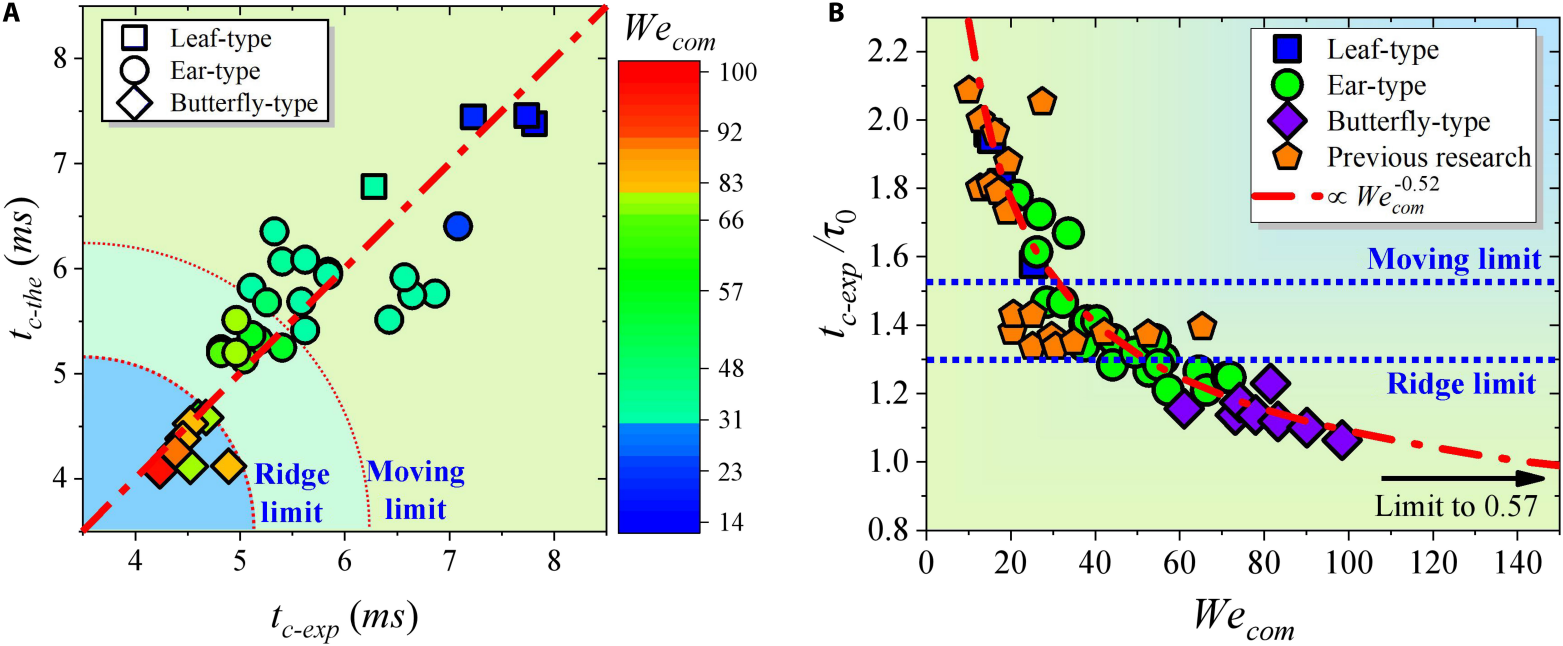
The relationship between theoretical contact time and experimental contact time. (A) Consistency relationship between experimental contact time and theoretical contact time based on 3 droplet impact behavior modes. (B) Inverse relation between the dimensionless experimental contact time *t_c-exp_*/*τ*_0_ and composite Weber number *We_com_*. The red line is the theoretical prediction as Eq. 8.

Combining the 3 types and the corresponding model assumptions, the theoretical contact time *t_c-the_* is obtained, which is highly consistent with the experimental values *t_c-exp_*, as shown in Fig. 8A. At the same time, the contact time of different types can be clearly distinguished. The contact time of Leaf-type is obviously higher than the theoretical limit contact time 1.56*τ*_0_ of moving surface. Meanwhile, the contact time of Butterfly-type is markedly shorter than the theoretical limit contact time 1.3*τ*_0_ for static ridge, and the contact time of Ear-type is between the 2 types. Further, both experimental results (Fig. 4A and B) and theoretical analysis show that *t_c_* is negatively correlated with *We_τ_* and *We_n_*. Therefore, we assume that *t_c_* has a certain quantitative relationship with the coupling of *We_τ_* and *We_n_*. Initially, the droplet has a vertical downward velocity *V* at the moment of contacting the ridge. At the same time, the droplet gets a tangential velocity *V_τ_* in the same direction as the surface movement affected by the moving surface. In order to reflect the influence of moving surfaces on the tangential velocity of the droplet, many related studies have been conducted, among which the restitution coefficient *ε_τ_* is favored as a dimensionless number [40–43]. The restitution coefficient *ε_τ_* is defined as the ratio of tangential velocity *V_τ_* at the moment of the droplet bouncing off the surface to the surface moving velocity *U*, i.e., *ε_τ_* = *V_τ_*/*U*. In our experiment, the restitution coefficient tends toward a constant value *ε_τ_* = 0.262 (see Fig. S5). Therefore, the composite velocity *V_com_* reflecting the velocity property of the droplet is represented as:

$$V_{\mathrm{com}}=\sqrt{V^{2}+V_{\tau }^{2}}=\sqrt{V^{2}+{\left(\varepsilon_{\tau }U\right)}^{2}}$$ (6)

Therefore, the corresponding composite Weber number *We*_com_ is expressed as:

$${\text{We}}_{\mathrm{com}}=\frac{\rho V_{\mathrm{com}}^{2}D_{0}}{\sigma }=\frac{\rho \left[V^{2}+{\left(\varepsilon_{\tau }U\right)}^{2}\right]D_{0}}{\sigma }={\text{We}}_{n}+\varepsilon_{\tau }^{2}{\text{We}}_{\tau }$$ (7)

Finally, the inverse relationship between dimensionless contact time *t_c-exp_*/*τ*_0_ and *We_com_* is discovered based on experimental data (see Fig. 8B). In Fig. 8B, the 3 types can be clearly distinguished by *We_com_*. Meanwhile, the best-fitting line based on the experimental data can be expressed as:

$$\frac{t_{c-\exp}}{\tau_{0}}=\frac{5.68}{{\text{We}}_{\mathrm{com}}^{0.52}}+0.57$$ (8)

Obviously, the inverse scaling law of −0.52 between *We_com_* and *t_c-exp_*/*τ*_0_ is illustrated, and the contact time is asymptotically close to 0.57*τ*_0_ with a 78% reduction rate when *We_com_* approaches positive infinity. The previous results [15,16,44] on the droplet impact on the stationary ridge surface also satisfy the scaling law (yellow dots in Fig. 8B). However, it has to be considered that the further increases of *We_com_* will lead to the droplet splash, and an analysis of this complex scenario is beyond the scope of current research so that the above model fails [25,45].

## Conclusion

Inspired by the surface structure of flying insects or wind-dispersal seeds, we have experimentally, numerically, and theoretically explored the mechanism of contact time reduction under the synergistic effect of moving and ridge surface. A synergistic 60% contact time reduction rate is achieved under the range of normal Weber number *We_n_* (14.7 ≤ *We_n_* ≤ 71.2) and the tangential Weber number *We_τ_* (0 ≤ *We_τ_* ≤ 403.5), which is the highest reduction rate among asymmetric rebound methods. The theoretical contact time model for the droplet morphology of each subdivision is established, which is consistent with the experimental contact time. Besides, the Leaf-type, Ear-type, and Butterfly-type modes of droplet impact on the moving ridge surface in the Weber numbers domain are revealed. Based on the dynamic behavior of the droplet, the contact time *t_c_* is divided into 4 main subdivisions: dropping time *t_d_*, spreading time *t_s_*, breaking time *t_b_*, and retraction time *t_r_*. In addition, we propose the composite Weber number (*We_com_*) in order to comprehensively consider the effect of *We_n_* and *We_τ_* on *t_c_*, and the inverse scaling law of −0.52 between *We_com_* and *t_c_* is illustrated. This new inverse relationship is highly in agreement with the contact time of both stationary ridge and moving ridge surface. This study lays a theoretical and experimental foundation for improving the application efficiency of bionic functional surfaces.

## Methods

### Experimental setup

Experiments were performed in ambient environment at room temperature with ~50% to 60% relative humidity. The apparatus was placed on a vibration isolation platform (POT-B). The light source uses 2 light-emitting diode lights (150 W/200 W with a color temperature of 5,500 ± 200 K) that are applied for illumination. Deionized water droplets with diameters *D*_0_ for 2.1 ± 0.05 mm were created by stainless steel needles, connected to a 10-ml plastic syringe and a syringe pump (LSP01-3A) via a rubber tube, from a predetermined height. The sample was placed on the center of a spin coater and absorbed to the base by vacuum. The angular velocity of the spin coater, changed from 300 to 1,200 rpm, can be directly adjusted at the control panel. Two high-speed cameras (Revealer, X213 with Nikon 105mmf/2.8G lens) were synchronized to capture the droplet impact dynamics from both side and top views at a frame rate of 13,698 frames per second. The images were analyzed by the software ImageJ.

### Sample fabrications

The superhydrophobic surface used in the experiment is Al-based substrate. First, the aluminum substrate was cut by machine tool to obtain the corresponding macroscopic ridge structure. Then, the polished aluminum sheet is cleaned with alcohol and immersed in deionized water for ultrasonic cleaning to obtain a clean surface. Subsequently, the surface is subjected to drying and cooling. After the completion of surface cleaning, the surface will undergo a superhydrophobic treatment by a commercial agent (NeverWet, Rust-Oleum Corporation, USA). First, the surface is evenly sprayed for 3 times with NeverWet-Step1, each with an interval of 20 to 30 min, to obtain a layer of adhesive surface layer. The surface layer is then sprayed 3 times with NeverWet-Step2, with an interval of 3 min. The NeverWet-Step2 spray was allowed to cure naturally in a windless environment, resulting in a uniformly hydrophobic surface.

### Characterizations

To characterize the surface wettability, the corresponding contact angle is extracted through image processing by taking several pictures of the liquid droplets resting on the superhydrophobic surface. The static contact angles were measured to be 163° ± 3°, illustrating excellent nonwetting property. Five independent measurements were carried out to obtain the average contact angles. The surface morphology of the samples was then observed with a scanning electron microscope at an accelerating voltage of 15 kV, and the surface morphology was obtained by collecting secondary electron images.

### Numerical methods

The numerical calculations were conducted in software COMSOL Multiphysics 6.1. The dynamics of water droplets impacting moving ridge surfaces were simulated using the coupled laminar and two-phase flow. Water and air were simulated within a rectangular domain with a length of 8.0 mm, a width of 10.0 mm, and a height of 5.0 mm, which contains more than 3.0 million unstructured tetrahedral cells. To enhance the accuracy of calculations, the near-wall grid was refined to include a multi-layer boundary layer. The corresponding display time interval, 1.0 μs, total 10.0 ms, is of high efficiency and convergence. The bottom surface was treated as a moving wall with different translational speeds, and the other surfaces of the rectangular domain were set as pressure outlet boundaries. As for the 2 phases, the primary one was set as air, while the secondary one was set as water. At the initial stage, the water droplet was modeled as a sphere with a downward impact velocity.

See the Supplementary Materials for more details about the impacting process and more data.

## Data Availability

The data that support the findings of this study are available from the corresponding author upon reasonable request.

## References

[1] Wang W, Yu W, Yu Z, Chen S, Cao D, Liu X, Zhao J. Non-axisymmetric bouncing dynamics on a moving superhydrophobic surface. Symmetry. 2023;16(1):29.

[2] Yu W, Zhu D, Wang W, Yu Z, Chen S, Zhao J. The rebounding-coalescing behaviors in drop-on-drop impact on a superhydrophobic surface. Appl Phys Lett. 2022;121:061602.

[3] Liu D, Tan H-W, Tran T. Droplet impact on heated powder bed. Soft Matter. 2018;14(48):9967–9972. 30499581 10.1039/c8sm01858h

[4] Damak M, Hyder MN, Varanasi KK. Enhancing droplet deposition through in-situ precipitation. Nat Commun. 2016;7:12560. 27572948 10.1038/ncomms12560 PMC5013560

[5] Ferrari M, Benedetti A. Superhydrophobic surfaces for applications in seawater. Adv Colloid Interf Sci. 2015;222:291–304. 10.1016/j.cis.2015.01.005 25759005

[6] Xiang Y, Huang S, Lv P, Xue Y, Su Q, Duan H. Ultimate stable underwater superhydrophobic state. Phys Rev Lett. 2017;119(13): Article 134501. 29341680 10.1103/PhysRevLett.119.134501

[7] Landel JR, Wilson DI. The fluid mechanics of cleaning and decontamination of surfaces. Annu Rev Fluid Mech. 2021;53:147–171.

[8] Vahabi H, Wang W, Mabry JM, Kota AK. Coalescence-induced jumping of droplets on superomniphobic surfaces with macrotexture. Sci Adv. 2018;4(11):eaau3488. 30430135 10.1126/sciadv.aau3488 PMC6226286

[9] Liu C, Zhao M, Zheng Y, Cheng L, Zhang J, Tee CATH. Coalescence-induced droplet jumping. Langmuir. 2021;37(3):983–1000. 33443436 10.1021/acs.langmuir.0c02758

[10] Kreder MJ, Alvarenga J, Kim P, Aizenberg J. Design of anti-icing surfaces: Smooth, textured or slippery? Nat Rev Mater. 2016;1(1):15003.

[11] Shen Y, Wu X, Tao J, Zhu C, Lai Y, Chen Z. Icephobic materials: Fundamentals, performance evaluation, and applications. Prog Mater Sci. 2019;103:509–557.

[12] Richard D, Clanet C, Quéré D. Contact time of a bouncing drop. Nature. 2002;417:811. 10.1038/417811a 12075341

[13] Bird JC, Dhiman R, Kwon H-M, Varanasi KK. Reducing the contact time of a bouncing drop. Nature. 2013;503(7476):385–388. 24256803 10.1038/nature12740

[14] Gauthier A, Symon S, Clanet C, Quéré D. Water impacting on superhydrophobic macrotextures. Nat Commun. 2015;6:8001. 26259509 10.1038/ncomms9001 PMC4918367

[15] Lin D-J, Wang L, Wang X-D, Yan W-M. Reduction in the contact time of impacting droplets by decorating a rectangular ridge on superhydrophobic surfaces. Int J Heat Mass Transf. 2019;132:1105–1115.

[16] Shen Y, Tao J, Tao H, Chen S, Pan L, Wang T. Approaching the theoretical contact time of a bouncing droplet on the rational macrostructured superhydrophobic surfaces. Appl Phys Lett. 2015;107:111604.

[17] Shu Y, Chu F, Hu Z, Gao J, Wu X, Dong Z, Feng Y. Superhydrophobic strategy for nature-inspired rotating microfliers: Enhancing spreading, reducing contact time, and weakening impact force of raindrops. ACS Appl Mater Interfaces. 2022;14(15):57340–57349. 36512411 10.1021/acsami.2c16662

[18] Lin C, Zhang K, Chen X, Xiao L, Chen S, Zhu J, Zou T. Reducing droplet contact time and area by craterlike surface structure. Phys Rev Fluids. 2021;6(8): Article 083602.

[19] Shen Y, Liu S, Zhu C, Tao J, Chen Z, Tao H, Pan L, Wang G, Wang T. Bouncing dynamics of impact droplets on the convex superhydrophobic surfaces. Appl Phys Lett. 2017;110.

[20] Aboud DG, Kietzig A-M. On the oblique impact dynamics of drops on superhydrophobic surfaces. Part II: Restitution coefficient and contact time. Langmuir. 2018;34:9889–9896. 29957965 10.1021/acs.langmuir.8b01233

[21] Biroun MH, Rahmati M, Tao R, Torun H, Jangi M, Fu Y. Dynamic behavior of droplet impact on inclined surfaces with acoustic waves. Langmuir. 2020;36:10175–10186. 32787026 10.1021/acs.langmuir.0c01628 PMC8010791

[22] Liu Y, Moevius L, Xu X, Qian T, Yeomans JM, Wang Z. Pancake bouncing on superhydrophobic surfaces. Nat Phys. 2014;10:515–519. 28553363 10.1038/nphys2980 PMC5444522

[23] Liu Y, Whyman G, Bormashenko E, Hao C, Wang Z. Controlling drop bouncing using surfaces with gradient features. Appl Phys Lett. 2015;107:051604.

[24] Moqaddam AM, Chikatamarla SS, Karlin IV. Drops bouncing off macro-textured superhydrophobic surfaces. J Fluid Mech. 2017;824:866–885.

[25] Zhan H, Lu C, Liu C, Wang Z, Lv C, Liu Y. Horizontal motion of a superhydrophobic substrate affects the drop bouncing dynamics. Phys Rev Lett. 2021;126(23): Article 234503. 34170170 10.1103/PhysRevLett.126.234503

[26] Tao R, Fang W, Wu J, Dou B, Xu W, Zheng Z, Li B, Wang Z, Feng X, Hao C. Rotating surfaces promote the shedding of droplets. Research. 2023;6:0023. 37040478 10.34133/research.0023 PMC10076004

[27] Zhang X, Zhu Z, Zhang C, Yang C. Reduced contact time of a droplet impacting on a moving superhydrophobic surface. Appl Phys Lett. 2020;117:151602.

[28] Wang M, Shi Y, Wang S, Xu H, Zhang H, Wei M, Wang X, Peng W, Ding H, Song M. Directional droplet bouncing on a moving superhydrophobic surface. Iscience. 2023;26(4):106389. 37013191 10.1016/j.isci.2023.106389 PMC10066525

[29] Gauthier A, Bouillant A, Clanet C, Quéré D. Aerodynamic repellency of impacting liquids. Phys Rev Fluids. 2018;3(5): Article 054002.

[30] Sawaguchi E, Matsuda A, Hama K, Saito M, Tagawa Y. Droplet levitation over a moving wall with a steady air film. J Fluid Mech. 2019;862:261–282.

[31] Yu F, Yang J, Tao R, Tan Y, Wang J, Wang D, Chen L, Wang Z, Deng X. Aerodynamic super-repellent surfaces. Research. 2023;2023:0111. 37223699 10.34133/research.0111 PMC10202376

[32] Shu Y, Hu Z, Feng Y, Wu X, Dong Z, Chu F. Prince Rupertʼs drop bouncing on high-speed moving superhydrophobic surfaces. Int Commun Heat Mass Transfer. 2023;148:107049.

[33] Wang M, Jiang Y, Gao P, Lu T, Lu J, Su T, Song M. Asymmetric deposition on high-speed moving superhydrophobic surfaces. J Mater Chem A. 2024;12(22):13086–13096.

[34] Zhang L-Z, Chen X, Yang Y-R, Wang X-D. Impact dynamics of a droplet on superhydrophobic cylinders structured with a macro ridge. Langmuir. 2023;39(18):6375–6386. 37092810 10.1021/acs.langmuir.3c00067

[35] Qian L, Huang C, Lv L, Fu Q, Fu C. Dynamic behavior of droplets impacting cylindrical superhydrophobic surfaces with different structures. Phys Fluids. 2023;35:023331.

[36] Hu Z, Chu F, Lin Y, Wu X. Contact time of droplet impact on inclined ridged superhydrophobic surfaces. Langmuir. 2022;38(4):1540–1549. 35072484 10.1021/acs.langmuir.1c03001

[37] Li D, Shang Y, Wang X, Zhang J. Dynamic behavior of droplet impacting on a moving surface. Exp Thermal Fluid Sci. 2024;153(2): Article 111126.

[38] Clanet C, Béguin C, Richard D, Quéré D. Maximal deformation of an impacting drop. J Fluid Mech. 2004;517:199–208.

[39] Bartolo D, Josserand C, Bonn D. Retraction dynamics of aqueous drops upon impact on non-wetting surfaces. J Fluid Mech. 2005;545:329–338.

[40] Aria AI, Gharib M. Physicochemical characteristics and droplet impact dynamics of superhydrophobic carbon nanotube arrays. Langmuir. 2014;30(23):6780–6790. 24866696 10.1021/la501360t

[41] Bertola V. An experimental study of bouncing Leidenfrost drops: Comparison between Newtonian and viscoelastic liquids. Int J Heat Mass Transf. 2009;52(7-8):1786–1793.

[42] Biance A-L, Chevy F, Clanet C, Lagubeau G, Quéré D. On the elasticity of an inertial liquid shock. J Fluid Mech. 2006;554:47–66.

[43] Chen L, Xiao Z, Chan PC, Lee Y-K, Li Z. A comparative study of droplet impact dynamics on a dual-scaled superhydrophobic surface and lotus leaf. Appl Surf Sci. 2011;257(1):8857–8863.

[44] Baggio M, Weigand B. Numerical simulation of a drop impact on a superhydrophobic surface with a wire. Phys Fluids. 2019;31:112107.

[45] Buksh S, Marengo M, Amirfazli A. Impacting of droplets on moving surface and inclined surfaces. Atomization Spray. 2020;30(8):557–574.

