# Supplementary material for: The Synergistic Reduction of the Contact Time in the Droplet Impact on a Moving Ridge Surface
Source: Research (Wash D C). 2024 Dec 9;7:0543. doi: 10.34133/research.0543 (PMC12327433; doi:10.34133/research.0543)
Supplement: Supplementary 1 — Figs. S1 and S5 Movies S1 to S3 [file research.0543.f1.zip › Supplementary Materials.docx]

**Fig. S1**

Comparison between theoretical impact velocity and measured impact velocity

**Fig. S2**

Variation of the normalized spreading contact time *t_s_*/*t_c_* as a function of *We_τ_*.

**Fig. S3**

Linear relationship between *D_τ_*/*D*_0_ and the function of *We_n_* and *We_τ_* from Damon et. al. [*On the Oblique Impact Dynamics of Drops on Superhydrophobic Surfaces*].

**Fig. S4**

Linear relationship between *D_τ_*/*D_n_* and the function of *We_n_* and *We_τ_* from Li et.al. [*Dynamic behavior of droplet impacting on a moving surface.*]

**Fig. S5**

Variation of the tangential velocity restitution coefficient *ε_τ_* as a function of *We_τ_*.

**Movie S1**

The evolutionary behavior of Leaf-type impact with *We_τ_*=11.2 and *We_n_*=14.7. From left to right are the behavioral evolution of experiments and simulations, and from top to bottom are the behavioral evolution in the front view and top view, respectively.

**Movie S2**

The evolutionary behavior of Ear-type impact with *We_τ_*=403.5 and *We_n_*=37.3. From left to right are the behavioral evolution of experiments and simulations, and from top to bottom are the behavioral evolution in the front view and top view, respectively.

**Movie S3**

The evolutionary behavior of Butterfly-type impact with *We_τ_*=100.9 and *We_n_*=71.2. From left to right are the behavioral evolution of experiments and simulations, and from top to bottom are the behavioral evolution in the front view and top view, respectively.
